# Orthorexia Nervosa Tendencies in Two Cohorts of Polish Young Adults: A Comparative Analysis of Prevalence, Correlates, and Comorbidity

**DOI:** 10.3390/nu17132208

**Published:** 2025-07-02

**Authors:** Izabela Łucka, Artur Mazur, Anna Łucka, Julia Trojniak, Marta Kopańska

**Affiliations:** 1 Department of Developmental Psychiatry, Psychotic Disorders and Old Age Psychiatry, Medical University of Gdansk, 80-210 Gdansk, Poland; izabelalucka@wp.pl; 2 Faculty of Medicine, University of Rzeszów, al. Tadeusza Rejtana 16C, 35-959 Rzeszów, Poland; drmazur@poczta.onet.pl; 3 Faculty of Law and Administration, University of Gdansk, 80-309 Gdansk, Poland; lucka.annaa@gmail.com; 4 Student Research Club “Reh-Tech”, Faculty of Medicine, University of Rzeszów, al. Tadeusza Rejtana 16C, 35-959 Rzeszów, Poland; juliatrojniak0@gmail.com; 5 Department of Medical Psychology, Faculty of Medicine, University of Rzeszów, al. Tadeusza Rejtana 16C, 35-959 Rzeszów, Poland

**Keywords:** orthorexia nervosa, ON, prevalence, eating disorders, smoking, ORTO-15, EAT-26, young adults, students, BMI, gender

## Abstract

**Background:** The rising focus on dietary choices has contributed to maladaptive eating patterns, including orthorexia nervosa (ON)—a pathological preoccupation with healthy eating. This study investigated ON prevalence and correlates in two Polish young adult cohorts to address inconsistencies in the existing literature and ON’s ambiguous nosological status. We explored its complex interplay with specific lifestyle and sociodemographic factors. **Methods:** The study sample consisted of 412 young adults, comprising Group 1 (G1; n = 136; 95 women, 38 men, and 3 non-binary individuals) and Group 2 (G2; n = 264; 194 women, 65 men, and 5 non-binary individuals). Data collection utilized a proprietary questionnaire for sociodemographic and health, the ORTO-15 questionnaire (cut-off < 35 points) for ON risk, and the EAT-26 for eating disorder (ED) risk. Depression was self-assessed. An analysis of sociodemographic, clinical, and lifestyle data was conducted to explore the association with orthorexia risk. **Results:** ON risk was identified in 26.5% of participants in G1 and 76.8% in G2. Logistic regression analysis identified different, independent predictors of ON risk for each group. In G1, these were depressive symptoms (OR = 2.52) and a co-occurring risk of eating disorders (ED) (OR = 11.37). In contrast, for G2, the predictors were smoking (OR = 2.14) and, inversely, a lower ED risk (OR = 0.16). No consistent associations were found with ON risk and age, gender, education, residence, or occupational status. **Conclusions:** This study confirms a strong link between ON and other eating disorders. The high ON prevalence in G2, combined with low internal consistency of ORTO-15, suggests tool limitations in specific populations. These findings highlight the need for more precise ON diagnostic tools and further research into its correlates, including body image, specific lifestyle factors, and its role within eating pathology.

## 1. Introduction

The increasing number of individuals experiencing the consequences of improper nutrition has prompted researchers worldwide to reflect on the genesis and nature of eating disorders, which have become a civilizational disease, especially in the so-called Western culture [1,2]. Since food not only aims to satisfy hunger by providing the body with an adequate number of calories but also influences emotional regulation and plays a role in social relationships, a range of additional phenomena are observed in individuals with underweight, obesity, and other eating disorders [1,2]. Among these is a pathological focus on healthy eating, first identified and described by S. Bratman in 1997 as orthorexia nervosa (ON) [3,4]. The prefix *“ortho-”* means “correct,” and *“orexis”* means “hunger, appetite.” The primary goal of individuals suffering from orthorexia, according to their declarations, is to achieve an ideal state of health through rigorous control of their diet. Individuals struggling with this disorder primarily focus on the quality of consumed products rather than their quantity, dedicating a significant amount of time to analyzing the origin of food, methods of its preparation, processing procedures, preservative content, and the type of materials used for packaging. An important element of orthorexia is also the tendency to hoard selected products, meticulously weigh and measure ingredients, carefully plan meals well in advance, and have persistent thoughts related to food during moments free from this activity [5]. The daily routine of patients is sometimes described as a four-stage process involving planning, ingredient selection, meal preparation, and final feelings of satisfaction or guilt [6].

Young adulthood represents a critical developmental period characterized by significant life transitions, identity formation, and the establishment of long-term health behaviors [7]. This phase is also associated with heightened vulnerability to mental health challenges, including eating disorders [8]. University students, in particular, constitute a high-risk population due to exposure to unique stressors such as academic pressure, new social environments, and increased autonomy, which can trigger maladaptive coping mechanisms like rigid dietary control [7]. Simultaneously, the broader population of young adults is heavily influenced by socio-cultural pressures, including the pervasive promotion of “healthism” and idealized body images on social media, making them susceptible to developing disordered eating patterns [9].

While both university students and the general young adult population are recognized as being at risk for ON, they exist in distinct life contexts that may shape the expression and correlates of the disorder differently. Students’ behaviors may be more influenced by the academic calendar, campus culture, and immediate peer groups, whereas other young adults may be more affected by factors related to occupational life, financial independence, and different sources of health information [7,9,10]. To date, few studies have directly compared these two subgroups within the young adult demographic to investigate these potential differences. A comparative approach is therefore crucial for determining whether population-specific prevention and intervention strategies are needed. This study aims to address this gap by directly comparing ON prevalence and its correlates in these two distinct Polish cohorts.

In light of the aforementioned context and to address the gap in comprehensive data concerning the differentiation of ON across various demographic groups in Poland, the following research hypotheses were formulated:

**H1:**  The frequency of orthorexia tendencies (defined based on the ORTO-15 scale score) will significantly differ between the studied populations of Group 1 and Group 2.

**H2:**  It is predicted that specific sociodemographic factors (such as age, gender, education level, and place of residence) and health-related behaviors (including smoking status, alcohol consumption, self-assessed presence of depression, and family history of eating disorders) will be associated with orthorexia tendencies. The strength and direction of these associations may differ across individual groups, reflecting their unique characteristics and life contexts.

**H3:**  In Group 1, due to the application of the EAT-26 scale, a strong association is expected between orthorexia tendencies and other symptoms of eating disorders, which will confirm the widely discussed overlap of these constructs in the literature.

Understanding these differences and factors associated with orthorexia is crucial for several strategic reasons. Firstly, it allows for a better understanding of ON epidemiology and the identification of particularly vulnerable groups, which is fundamental for planning targeted public health prevention and educational activities. Secondly, identifying specific correlates in different populations can provide valuable guidance for clinicians, psychologists, and healthcare professionals, supporting early detection and the development of more personalized intervention strategies. Finally, a comprehensive analysis of data from such diverse groups contributes to enriching the general knowledge about the nature of orthorexia itself, its manifestations in different demographic and social contexts, which can support future work on improving diagnostic tools and classification criteria.

## 2. Materials and Methods

### 2.1. Study Participants

The study was conducted on two independent groups of respondents from Poland. All participants voluntarily participated in the study and were informed about its purpose and the anonymity of the collected data, in accordance with the principles of the Declaration of Helsinki. Consent to participate in the study was obtained from all participants prior to completing the questionnaires. The study protocol was approved by the Ethics Committee for Scientific Research of the University of Gdansk (protocol code 60/2024/WNS of 11 October 2024).

Group 1 (G1) consisted of 136 participants, including 95 females (69.9%), 38 males (27.9%), and 3 individuals identifying as non-binary (2.2%). This group included students and young adults from various fields of study, mostly not directly related to health.

Group 2 (G2) included 276 adult individuals, with 203 females (73.6%), 68 males (24.6%), and 5 individuals identifying as non-binary (1.8%). This group included adults and young adults.

### 2.2. Characteristics and Rationale for Group Selection

The study involved two distinct cohorts of Polish young adults, recruited independently. While preliminary analysis showed the groups were nearly identical in mean age (G1: 23.2 ± 3.2 vs. G2: 23.3 ± 4.7 years) and proportion of individuals with higher education (G1: 71.3% vs. G2: 72.0%), a more detailed analysis revealed significant differences in their occupational profiles and dietary behaviors, providing a strong rationale for their comparison.

Group 1 (G1; n = 136) represents a cohort of young adults with a mixed life context. While a significant portion were students (43.4% reporting “Student” as their primary status), this group also included a larger contingent of individuals primarily active in the workforce (28.7% reporting “Working” only).

Group 2 (G2; n = 276) can be characterized as a cohort more deeply embedded in the academic environment. This group had a significantly higher proportion of participants who were solely students (55.1%) and a smaller proportion of those who were only working (19.9%).

This difference in life context was mirrored by a stark contrast in dietary habits. Based on an analysis of self-reported data:

In Group 1, 33.1% of participants reported currently or previously following a special diet (e.g., vegetarian, vegan, gluten-free).

In Group 2, this figure was substantially higher, with 54.3% of participants reporting the use of such diets.

The comparison of these two groups is therefore justified not by age or education, but by these crucial differences in life context (work vs. academic focus) and health-related behaviors (prevalence of special diets). This design allows for exploring how these distinct contextual factors relate to orthorexia nervosa tendencies. A summary of these key differentiating characteristics is presented in Table 1.

### 2.3. Research Tools

A set of questionnaires was used in both groups to assess the risk of orthorexia, eating disorders, and to gather demographic data and information on potential correlates: 

**Proprietary Questionnaire:** This was used to collect sociodemographic data (gender, age, place of residence, level of education, professional status), information on health status, body weight and height (to calculate BMI), as well as data concerning lifestyle, including the use of psychoactive substances and the occurrence of mental disorders (including depression and eating disorders) in the family. 

**ORTO-15:** To assess orthorexia nervosa (ON) tendencies, the Polish adaptation of the ORTO-15 questionnaire, originally developed by Donini et al., was used [3,11]. The Polish validation was conducted by Stochel, Janas-Kozik et al. [12]. The questionnaire consists of 15 items rated on a 4-point Likert scale (“always,” “often,” “sometimes,” “never”). These items assess cognitive, clinical, and emotional aspects of an obsessive approach to healthy eating. The total score ranges from 15 to 60 points, where a lower score indicates a higher risk of ON Based on the Polish validation study and previous research, a cut-off point of <35 was adopted as indicative of an increased risk of orthorexia nervosa. The internal consistency of the ORTO-15 scale was assessed using Cronbach’s Alpha coefficient for the studied groups to verify the reliability of the tool in the context of the analyzed populations—Table 2.

The authors acknowledge that ORTO-15 measures a continuum of orthorexia symptoms rather than a definitive diagnosis. Despite these limitations, its use in this study allows for comparison of results with earlier publications and contributes to the global accumulation of data on this phenomenon. 

**Eating Attitudes Test (EAT-26):** To screen for the risk of eating disorders (ED), the Polish version of the EAT-26 questionnaire was used, validated by Rogoza et al. [13]. The original EAT-40 scale was developed by Garner and Garfinkel in 1982, from which the 26-item version (EAT-26) was derived [14]. The tool consists of 26 items rated on a 6-point Likert scale, assessing attitudes and behaviors related to eating. The EAT-26 is composed of three subscales: “Dieting,” “Bulimia and Food Preoccupation,” and “Oral Control”. A higher total score indicates greater severity of symptoms characteristic of ED. A score of 20 or more is used as a cut-off to classify an individual into the group at risk for an eating disorder.

**Assessment of Depressive Symptoms:** Information about depression was collected via self-assessment (a single question: “Do you suffer from depression? Yes/No” or regarding a diagnosis). The authors acknowledge that the use of a single, non-validated question is a limitation of the study. However, this method has been examined as a brief case-finding instrument in other settings. For instance, a comprehensive meta-analysis by Mitchell et al. found that a single question asking about depression can have acceptable sensitivity (approximately 82%) for screening purposes, although its specificity is lower [15]. This method was therefore considered sufficient for an initial, exploratory assessment of self-perceived depressive tendencies in the context of this study. 

**Seeking Professional Mental Health Assistance:** Participants were also asked about their history of seeking professional mental health support (psychologist, psychiatrist, or psychotherapist). This question was designed to gather basic information about clinical history, which could be an indication of whether the participants perceived mental health problems, modeled on items used in large-scale public health and epidemiological surveys assessing mental health service utilization, such as the World Health Organization (WHO) World Mental Health Surveys [16].

**Body Mass Index (BMI):** Calculated based on self-reported body weight (in kg) and height (in m) by participants, according to the formula BMI = weight/(height)^2^. The obtained values were categorized according to World Health Organization (WHO) criteria: Underweight (BMI < 18.5), normal weight (18.5–24.9), overweight (25.0–29.9), and obesity (BMI ≥ 30.0)—combined into one category (≥25.0) [17].

**Psychoactive Substance Use:** Assessed based on questions from the proprietary questionnaire regarding the frequency and type of substances used:

- **Tobacco Smoking:** Participants were asked to define their smoking status using the categories: “I am a smoker,” “I am a former smoker,” or “I have never smoked.”
- **Alcohol Consumption:** Frequency of alcohol use was assessed with the question “How often do you consume alcohol?” with the following response options: “Never or a few times a year,” “1–3 times a month,” “1–2 times a week,” or “More than twice a week.” These categories are consistent with screening frameworks used in public health research to identify consumption patterns.
- **Illicit Drug Use:** Lifetime and recent use of illicit drugs was assessed with the question “Have you ever used drugs?” with response options including: “Never,” “A few times in my life,” “1–3 times a month,” “1–2 times a week,” or “More often.”

### 2.4. Study Procedure

Before participating in the study, participants were informed about its purpose, the voluntary nature of their involvement, and the assurance of anonymity. Consent was a prerequisite for participation. In G1 and G2, an online survey platform was used. Participants for both cohorts were recruited using a non-probabilistic, convenience sampling method conducted via online channels.

The recruitment for Group 1 was carried out between July 2023 and August 2024. The online survey link was disseminated through two primary channels: (1) targeted posts on social media platforms, specifically Facebook groups dedicated to Polish university students across various fields of study, and (2) direct email invitations.

A comparable strategy was employed for Group 2 throughout the year 2023. Recruitment in this cohort targeted a broader demographic of young adults, with the survey link distributed in online communities and forums focused on lifestyle, health, and well-being.

For both cohorts, this initial recruitment was supplemented by snowball sampling, as participants were encouraged to share the survey link within their own social and professional networks.

#### Inclusion and Exclusion Criteria

The inclusion criteria for participation in the study were established as follows:

- A minimum participant age of 17 years.
- Residency in Poland at the time of the survey.
- Fluency in the Polish language, in which all research instruments were administered.
- Provision of informed consent to participate in the study.

The only exclusion criterion was the submission of a substantially incomplete questionnaire, which would prevent the valid calculation of scale scores and subsequent statistical analysis.

### 2.5. Bioethical Committee Approval

The study was conducted in accordance with the principles of the Declaration of Helsinki. The project received approval from the Ethics Committee for Scientific Research at the University of Gdansk (approval no. 60/2024/WNS dated 11 October 2024). 

### 2.6. Statistical Analysis

Analyses were performed using IBM SPSS Statistics (version 27) and Statistica (version 13.1) software. To characterize the study groups (demographics, anthropometry, basic clinical features), descriptive statistics such as counts and percentages for categorical variables, and means, standard deviations (SD), and ranges for continuous variables were employed. The normality of variable distribution was checked using the Shapiro–Wilk test. Due to the failure to meet the assumption of normal distribution for most variables, non-parametric tests were used for intergroup comparisons (e.g., ON+ vs. ON−): the Mann–Whitney U test for the two groups. The internal consistency of the ORTO-15 scale in the groups was assessed using Cronbach’s Alpha coefficient, which is crucial for evaluating the reliability of the measure in the context of the studied populations. Chi-square (χ^2^) tests were used to compare the frequency of orthorexia tendencies (ORTO-15 < 35) in individual groups and the associations between categorical variables (e.g., gender, smoking status, depression status, and ON risk). Cramer’s V coefficient was reported as a measure of effect size for these tests. Relationships between ordinal or quantitative variables not meeting the normality condition were assessed using Spearman’s rank correlation coefficient (rho). For all reported percentages (e.g., ON prevalence), 95% Confidence Intervals [95% CI] were calculated to enhance the interpretation of practical significance. Similarly, for Pearson’s r correlation coefficients, 95% CI were provided. In cases of statistically insignificant results, *p*-values and corresponding effect sizes were also provided to allow for full interpretation. To identify the independent predictors of orthorexia nervosa (ON) risk and eating disorder (ED) risk, a series of multivariable binary logistic regression models was performed. A stratified approach was employed, running separate models for each study group. For the ON risk models, the dependent variable was the binary ON risk category (ORTO-15 score < 35), and the independent variables included group membership, age, gender, BMI, smoking status, depressive symptoms (BDI score or self-assessment), and the binary ED risk category (EAT-26 score ≥ 20). For the ED risk models, the dependent variable was the binary ED risk category, with the ON risk included as an independent variable. For all statistical analyses, a *p*-value of <0.05 was considered to indicate statistical significance.

## 3. Results

### 3.1. Characteristics of Study Groups

The sociodemographic, anthropometric, and selected health-related characteristics of the study groups (Group 1; Group 2) are detailed in Table 3. A total of 412 individuals participated in the study (G1: n = 136, G2: n = 276). Preliminary analysis revealed significant differences in the distribution of age, education level, and occupational status between the groups, reflecting their specific characteristics and social contexts. These differences will be discussed in the context of the observed results regarding orthorexia tendencies.

### 3.2. Prevalence of Orthorexia Nervosa (ON) and Eating Disorder (ED) Risk

The analysis of the prevalence of orthorexia risk (ON), measured using the ORTO-15 scale (score < 35 points), and eating disorder (ED) risk, determined based on the EAT-26 score, revealed significant variation between the study groups, as presented in Table 4.

ON risk was 26.5% in Group 1. In contrast, a dramatically higher percentage of individuals with ON risk was observed in Group 2, at 76.8%. This significant difference in the prevalence of ON risk between G1 and G2 is a key finding of the study. ED risk also showed variation between the groups, with 15.4% in G1 and 20.7% in G2.

ON risk in Group 1 was 26.5%, while in Group 2 it was significantly higher at 76.8%. This difference was statistically significant (*p* < 0.001). ED risk was 15.4% in Group 1 and 20.7% in Group 2; however, this difference was not statistically significant (*p* = 0.197).

### 3.3. Independent Predictors of ON and ED Risk

To identify the independent predictors for orthorexia (ON) risk and eating disorder (ED) risk, a multivariable logistic regression analysis was conducted separately for Group 1 and Group 2. The results are summarized in Table 5 and Table 6.

#### 3.3.1. Predictors of Orthorexia (ON) Risk

In Group 1 (n = 136), the presence of depression and the risk of an eating disorder (ED) were found to be significant, independent predictors of an increased ON risk. The presence of depressive symptoms increased the odds of ON risk by over 2.5 times (OR = 2.519; *p* = 0.040) compared to individuals without depression. An even stronger predictor was ED risk—individuals in this group had over 11 times higher odds of co-occurring ON risk (OR = 11.370; *p* < 0.001).

In Group 2 (n = 276), different predictors were identified. Smoking more than doubled the odds of ON risk (OR = 2.143; *p* = 0.028). Interestingly, ED risk in this group was an inverse predictor—individuals with eating disorder risk had approximately 84% lower odds of being diagnosed with ON risk (OR = 0.162; *p* < 0.001)—Table 5.

#### 3.3.2. Predictors of Eating Disorder (ED) Risk

The analysis of predictors for ED risk in Group 1 (n = 136) showed that gender, BMI, and ON risk were significant factors. Men had approximately 85% lower odds of ED risk compared to women (OR = 0.166; *p* = 0.032). Each one-point increase in BMI increased the odds of ED risk by 11.5% (OR = 1.115; *p* = 0.042). However, the strongest predictor was ON risk, which increased the odds of ED risk by over 12 times (OR = 12.149; *p* < 0.001).

In Group 2 (n = 276), gender, BMI, and ON risk were also significant predictors, but the direction and magnitude of these associations were different. In this cohort, the odds of ED risk for men were almost 11 times higher than for women (OR = 10.943; *p* < 0.001). A one-point increase in BMI was associated with a 9.8% decrease in the odds of ED risk (OR = 0.902; *p* = 0.009). Similar to the ON prediction model, ON risk in this group was an inverse predictor, decreasing the odds of ED risk by approximately 85% (OR = 0.148; *p* < 0.001)—Table 6.

### 3.4. Association Between ON Risk and ED Risk

Analysis of the relationship between orthorexia risk (ON) and eating disorder risk (ED) revealed statistically significant associations with varying direction and strength across the individual groups, as detailed in Table 7.

In Group 1 (students, mostly from fields not directly related to health), a strong association was demonstrated, with an OR of 11.190 [95% CI: 3.883; 32.247] (χ^2^(1) = 25.790; *p* < 0.001; V = 0.435). These results indicate a significantly increased risk of ON in individuals with ED risk in this group. 

A divergent trend was observed in Group 2 (adults). In this cohort, ON risk was less probable in individuals with ED risk (OR = 0.165 [95% CI: 0.088; 0.312]; χ^2^(1) = 35.0; *p* < 0.001; V = 0.356). This unique relationship for Group 2 requires more in-depth interpretation in the discussion section, taking into account the specificity of the studied population.

### 3.5. ON Risk and Body Mass Index (BMI)

The association between ON risk and BMI was consistent in the study groups. In G1, no significant difference in mean BMI was observed between the group with ON risk (22.07 ± 4.78) and the group without risk (23.06 ± 4.50) (Mann–Whitney U = 1673.5; *p* = 0.533). No significant correlation was found between ORTO-15 score and BMI (Spearman’s rho = 0.042, *p* = 0.626). In G2, the analysis of the association between ON risk and belonging to an abnormal body weight category (underweight, overweight, obesity) also did not show statistical significance (χ^2^ = 0.266, *p* = 0.606).

### 3.6. ON Risk and Depression

The assessment of the association between ON risk and depressive symptoms was hindered by the use of self-assessment in both G1 and G2 and yielded varied results—Table 8.

In Group 1, a statistically significant association was found between ON risk and self-reported depressive symptoms (χ^2^(1) = 3.927; *p* = 0.048; V = 0.170). Individuals with ON risk in G1 more frequently reported depression (37.0%) compared to those without ON risk (21.1%), with an OR of 2.191 [95% CI: 1.000; 4.798]. In Group 2, the association between ON risk and self-reported depressive symptoms was not statistically significant (χ^2^(1) = 3.498; *p* = 0.061; V = 0.113). In this group, 70.4% of individuals with ON risk reported depression, while 80.3% of those without ON risk reported depression, with an OR of 1.717 [95% CI: 0.971; 3.036].

### 3.7. ON Risk and Psychoactive Substance Use

Analysis of the association between ON risk and psychoactive substance use showed a significant result only for tobacco smoking in Group 2—Table 9.

#### 3.7.1. Tobacco Smoking

In Group 1, the association between ON risk and tobacco smoking was not statistically significant (χ^2^(1) = 1.054; *p* = 0.305; V = 0.088). However, in Group 2, a statistically significant association was noted (χ^2^(1) = 4.333; *p* = 0.037). In this cohort, while univariate analysis indicated a higher ON risk in non-smokers, although the strength of this association was small (Cramer’s V = 0.125), the multivariate model, after controlling for other variables, showed that smoking was an independent risk factor.

#### 3.7.2. Alcohol Consumption

In the case of regular alcohol consumption, no statistically significant associations with ON risk were observed in Groups 1 and 2. In Group 1, statistical analysis showed χ^2^(1) = 0.583; *p* = 0.445; V = 0.065. Similarly, in Group 2, χ^2^(1) = 0.006; *p* = 0.937; V = 0.005 was noted.

#### 3.7.3. Drug Use

Analyses concerning the association of ON risk with drug use in Groups 1 and 2 did not show statistical significance. In Group 1, χ^2^(1) = 3.744; *p* = 0.053; V = 0.166 was noted, where the *p*-value was close to the significance threshold; however, this result was based on a very small number of users. In Group 2, the analysis showed χ^2^(1) = 2.665; *p* = 0.103; V = 0.098.

### 3.8. ON Risk and Demographic Factors

The analysis of associations between orthorexia nervosa (ON) risk and demographic factors, such as age, gender, education level, place of residence, and occupational status, was conducted for each of the studied groups. Data regarding family factors in the context of ON risk were not available in this study.

#### 3.8.1. Age

In Group 1 (G1), the analysis showed no significant differences in age depending on ON risk (Mann–Whitney U = 2176; *p* = 0.887). Also, in Group 2 (G2), age was not significantly associated with ON risk (Mann–Whitney U = 15,840.5; *p* = 0.757).

#### 3.8.2. Gender

The association between gender and ON risk was not statistically significant in all groups. In Group 1 (G1), the analysis showed no significant differences (χ^2^(2) = 1.054; *p* = 0.305; V = 0.088). Analogously, in Group 2 (G2), the association of gender with ON risk was also not statistically significant (χ^2^(2) = 0.635; *p* = 0.728; V = 0.048).

#### 3.8.3. Level of Education

No statistically significant associations were noted between education level and ON risk in the available groups. In Group 1 (G1), the analysis showed no significant differences (χ^2^(2) = 0.583; *p* = 0.747; V = 0.065). Similarly, in Group 2 (G2), this association was not statistically significant (χ^2^(2) = 0.006; *p* = 0.997; V = 0.005).

#### 3.8.4. Place of Residence

The association between place of residence and ON risk was not statistically significant in Groups 1 (G1) and 2 (G2). In Group 1 (G1), the analysis showed no significant differences (χ^2^(2) = 0.583; *p* = 0.747; V = 0.065). In Group 2 (G2), this association was also not significant (χ^2^(2) = 0.006; *p* = 0.997; V = 0.005).

#### 3.8.5. Occupational Status

Occupational status did not show statistically significant associations with ON risk in Groups 1 (G1) and 2 (G2). In Group 1 (G1), the analysis showed no significant differences (χ^2^(3) = 3.744; *p* = 0.29; V = 0.166). In Group 2 (G2), this association was also not statistically significant (χ^2^(3) = 2.665; *p* = 0.446; V = 0.098).

### 3.9. ON Risk and Seeking Professional Mental Health Assistance

In the studied groups, the association between orthorexia risk (ON risk) and seeking psychological or psychiatric assistance was analyzed. The results are presented in Table 10.

In Group 1, statistical analysis showed no significant association between the presence of orthorexia and seeking psychological or psychiatric assistance (χ^2^ = 0.635, *p* = 0.426). Similarly, in Group 2, no significant statistical dependence was observed (*p* = 0.305) regarding ON risk and seeking specialized mental health assistance. The Odds Ratios (ORs) were 1.265 [95% CI: 0.709; 2.257] for Group 1 and 1.5007 [95% CI: 0.687; 3.306] for Group 2. The strength of the association, measured by Cramer’s V, was 0.048 for Group 1 and 0.088 for Group 2.

## 4. Discussion

This study aimed to compare the prevalence of orthorexia nervosa (ON) risk and its selected correlates and identify independent predictors of ON risk in two distinct young adult populations in Poland: Group 1, university students not directly involved in health care, and Group 2, young adults/adults. The selection of these heterogeneous research groups was driven by the need to explore the phenomenon of ON in various socio-educational contexts, which served as the basis for formulating three main hypotheses. The use of logistic regression models made it possible to identify independent predictors of orthorexia. The most important finding is that the set and nature of these predictors differ drastically between the studied groups. This indicates the heterogeneity of the phenomenon of orthorexia and confirms the validity of comparing both cohorts.

Firstly, it was hypothesized that the prevalence of orthorexia tendencies (H1) would significantly differ across the studied populations of G1 and G2, a premise derived from the hypothetical variation in exposure to risk factors for ON development in diverse social and educational environments. Secondly, it was predicted that specific sociodemographic factors and health-related behaviors (H2) would be associated with orthorexia tendencies, and the strength and direction of these associations might vary across individual groups, reflecting their unique characteristics. Finally, in G1 and G2, given the use of the EAT-26 scale, a strong association was hypothesized between orthorexia tendencies and other symptoms of eating disorders (H3), which was intended to confirm the widely discussed overlap of these constructs in the literature. The presented analysis provides valuable cross-sectional data, enabling a preliminary assessment of the phenomenon’s scale and the identification of potential factors associated with ON risk in these specific subpopulations, which is crucial for directing further research and preventive actions.

A key and most problematic finding of this study is the dramatic increase in the prevalence of ON risk in Group 2 (76.8%) compared to Group 1 (26.5%). The prevalence of ON risk in Group 1 using the <35 points threshold is consistent with some studies in the Polish population, e.g., the study by Stochel et al. validating ORTO-15, which suggested the adequacy of this threshold [12,18,19,20].

However, these rates are higher than in many international studies using the same threshold in student populations, where ON prevalence rarely exceeds 10–20% [4,21]. Conversely, they are significantly lower than estimates based on the <40 threshold, which, according to a meta-analysis by McComb and Mills, can average as high as 57.6% in general samples, confirming the rationale for choosing a more conservative <35 points threshold to avoid overdiagnosis [22]. For example, an extensive meta-analysis by López-Gil et al. estimated the average global prevalence of ON risk at the <35 points threshold to be 27.5%. Even in the highest-risk groups, such as athletes or individuals engaged in fitness training, this prevalence averaged 34.5% [23]. Also, a review of studies on athletes conducted by Paludo et al. suggests that average ORTO-15 scores in this group range from 35 to 38 points, indicating a much lower prevalence of risk according to the <35 points threshold than observed in our G2 (76.8%) [24]. Such an extremely high percentage may be inflated or misleading and requires thorough analysis.

On the other hand, the influence of specific characteristics of Group 2 cannot be excluded. As sociodemographic data indicate, Group 2 consisted of adults with a higher level of education (72.0% with higher education) and a larger percentage of residents of large cities (70.7%). The literature indicates that ON prevalence may differ between subgroups, e.g., depending on the field of study or profession [25,26]. Firstly, a higher level of education and residence in an urban environment could have contributed to increased health awareness and access to information on nutrition and pro-health trends. Studies indicate that orthorexia symptoms may be more common among individuals with a higher level of education and university students [9]. Urban environments often offer wider access to specialized food products and the literature on healthy lifestyles, and are places of more intensive information flow, including via social media, which may promote the development of orthorexia behaviors [27]. An increased amount of health information, if not critically processed, can lead to the adoption of more rigid and inflexible dietary rules [9]. Group 2, as young adults, although slightly older than typical undergraduate students, is still in a life period where habits are formed, and there is high susceptibility to social influences regarding health and appearance [28]. Some studies indicate very high prevalence rates of orthorexia tendencies in specific student groups, suggesting that certain subgroups of young adults, perhaps those more focused on achievement or self-improvement (which may be associated with higher education), may exhibit stronger such tendencies. The process of higher education itself may also be associated with stress and pressure, which, in some cases, can lead to seeking control in other areas of life, such as diet [29].

The high percentage of individuals with orthorexia tendencies in Group 2 may also be related to the phenomenon of “healthism” and personality traits such as perfectionism. “Healthism” is defined as the belief that health is a paramount value, and its maintenance through appropriate dietary choices becomes a personal responsibility and a measure of worth [9]. Such an attitude, combined with perfectionism—a trait often associated with orthorexia and potentially more common in individuals achieving higher levels of education—can lead to an obsessive focus on the quality and “purity” of food [9,27,30]. Meticulous meal planning, elimination of entire food groups, and strict adherence to dietary rules, observed in orthorexia, are consistent with the pursuit of perfection and the need for control. It is possible that the unique characteristics of G2 contributed to obtaining such high scores on this particular measurement tool. The high frequency of responses indicating orthorexia tendencies to individual ORTO-15 questions in this group seems to partially confirm this, although the scale of the phenomenon still raises doubts about the adequacy of the <35 points threshold in this population. Undoubtedly, further research on the validity and reliability of ORTO-15 and the search for alternative methods for ON diagnosis are needed.

As the literature indicates, the ORTO-15 questionnaire faces criticism regarding its validity and psychometric stability, and its interpretation can be problematic [31]. Systematic reviews confirm high variability in ON prevalence estimates depending on the tool, threshold, and studied population, suggesting that ORTO-15 may have difficulty distinguishing pathology from intense, normative interest in a healthy lifestyle, especially in certain groups [30]. This result might reflect a more general interest in health rather than a disorder [32].

Regardless of the reasons for such a high prevalence in Group 2, a consistent finding across both study groups is a strong association between ON risk and eating disorder (ED) risk. In Group 1, the multivariate analysis confirmed a very strong, positive association between the risk of ON and the risk of ED. This aligns with the prevailing view in the literature, supported by reviews and meta-analyses, that ON shares many features with anorexia and bulimia and should be classified within the eating disorder spectrum [29,33]. A much more complex picture emerges from the analysis of Group 2, where the regression model revealed a strong but inverse relationship—the risk of ED was associated with 84% lower odds of ON risk. This finding, as a result of a multivariate analysis, cannot be treated as a simple statistical artifact. It suggests that in this specific cohort, characterized by a very high prevalence of ON, the ORTO-15 scale may be capturing the phenomenon of ‘healthism’ and an intense, normative interest in health, which does not overlap with the pathology measured by the EAT-26. Understanding these interconnections is crucial for developing effective prevention strategies, as meta-analyses of eating disorder prevention programs have shown positive effects on improving knowledge and reducing maladaptive eating attitudes and behaviors, particularly when targeting higher-risk individuals [34].

The relationship between orthorexia nervosa and the obsessive–compulsive spectrum remains a subject of debate. While the international literature often suggests a link, findings from the Polish population indicate this relationship may be less significant than often assumed. Notably, in a previous large-scale study on over 800 Polish youths, co-authored by one of the authors of the present paper, Łucka et al., no significant correlation was found between ON risk (measured by ORTO-15) and general obsessive–compulsive symptoms (measured by MOCI) [19]. That study concluded that in this demographic, ON is more closely aligned with the eating disorder spectrum than with OCD. The current study, therefore, focused on deepening the analysis of ON’s connection to the ED spectrum and other behavioral correlates, which seemed a more promising direction based on prior local evidence.

Through the application of multivariate statistical analysis, our findings regarding depression also gained precision. The multivariate analysis confirmed that in Group 1, depression is not only correlated with ON but also constitutes its independent predictor, increasing the risk more than 2.5-fold. This reinforces the hypothesis of a significant role for affective psychopathology in the development of ON in this group. Importantly, in the model for Group 2, depression did not emerge as an independent predictor, suggesting that in this population, which has a different profile, other factors play a dominant role.

In Group 1, individuals with ON risk more frequently reported depression than those without risk. In contrast, in Group 2, this association was weaker and not statistically significant. This ambiguity, in addition to the potential influence of group specificity, is certainly partly related to the use of self-assessment, which constitutes a significant comparative limitation. Although in Group 2, this association did not achieve formal statistical significance, this value, being close to the significance threshold, may suggest a weaker but still present tendency for a relationship between ON and depression. This result might not have achieved significance due to lower statistical power or the influence of specific characteristics of group G2. This contrasts with the results of a prospective study conducted by Messer et al., which showed that a higher severity of ON symptoms significantly predicted an increase in depressive symptoms within three months. Both studies indicate the existence of an association between ON and depression; however, the prospective approach in the Messer et al. article is crucial because it allows for inferring that ON may be a risk factor or precursor for the development of depressive symptoms [35]. In our study, due to its cross-sectional nature, we are unable to determine the direction of these causal relationships. Nevertheless, data from the literature emphasize that affective psychopathology is an important element of the clinical picture associated with orthorexia. A study by Awad et al., for example, showed that depression, anxiety, and stress play a significant mediating role in the relationship between impulsivity and orthorexia tendencies, both in the pathological orthorexia nervosa dimension and the “healthy orthorexia” (HO) dimension [36]. This indicates that mood disorders are intertwined with the psychological mechanisms underlying or co-occurring with orthorexia. Furthermore, other studies identify traits strongly associated with depression risk as significant predictors of ON. Systematic reviews confirm a strong link between orthorexia tendencies and perfectionism [37,38]. Moreover, perfectionism has been shown to play a moderating role in the relationship between ON symptoms and obsessive–compulsive symptoms, and perfectionism itself is a recognized transdiagnostic factor linked, among others, to depression [38]. These data, indicating associations between ON and traits such as perfectionism, underscore the clinical importance of a comprehensive assessment of mental status, including potential depressive symptoms, in individuals with orthorexia tendencies.

The relationship between orthorexia tendencies (ON) and Body Mass Index (BMI) in our study proved to be complex and multifaceted. In the univariate analyses, we found no significant association between the risk of ON and BMI in either group. This would suggest that in the studied populations, orthorexia motivations might stem more from ‘healthism’ or perfectionism, rather than necessarily from a desire to change body weight [29]. Some studies, similar to our univariate analysis’s findings, show no association between ON and BMI, while others suggest a negative correlation, indicating lower BMI in individuals with higher orthorexia tendencies, especially when scales other than ORTO-15 are used (e.g., DOS) or in specific clinical groups, such as patients with AN/BN [39,40,41]. Analyses conducted in specific Polish populations, such as flight personnel, also provide data on this complex relationship [42]. In groups 1 and 2, where this association was not significant, the motivations for orthorexia may have other underlying causes, such as a more ingrained ‘healthism’ or perfectionism, not directly related to the initial goal of weight loss. Overall, these results suggest that the association between ON and BMI is weak and likely modified by other factors, such as the study population, ON measurement methods, or the stage of advancement of orthorexia tendencies.

However, a much more nuanced picture emerged from the multivariable logistic regression models, where BMI was found to be a significant and independent predictor of eating disorder (ED) risk, acting in opposite directions in the two cohorts.

Our finding for Group 1, where a higher BMI was a predictor of higher ED risk, is consistent with numerous international studies. For instance, a study by Abdalla et al. on a population of Malaysian students also found a significant positive correlation between EAT-26 scores and BMI, suggesting that a higher BMI is associated with a greater risk of abnormal eating attitudes [43]. This confirms that higher body mass is a recognized risk factor for the development of body dissatisfaction and restrictive behaviors. Furthermore, systematic reviews and meta-analyses, such as the work by Ralph-Nearman et al., indicate a bidirectional relationship where not only can a higher BMI predict the development of eating pathology, but symptoms of eating disorders can also lead to future changes in body mass [44]. In contrast, the result from Group 2 is surprising and requires a more in-depth interpretation. Here, an inverse relationship was observed: each one-point increase in BMI was associated with a 9.8% decrease in the odds of ED risk. This counterintuitive finding may stem from the specific characteristics of this cohort, which is marked by an extremely high prevalence of orthorexia tendencies. It can be hypothesized that in this group, which is steeped in ‘healthism,’ the pathological attitudes measured by the EAT-26 are most strongly expressed in individuals with normal or low body weight, for whom the perfectionistic pursuit of an ‘ideal’ body is a primary goal. Individuals with a slightly higher BMI within the same group might have a more relaxed approach or may not fit the psychological profile that leads to a high score on the EAT-26.

Although BMI did not emerge as a direct predictor of ON risk, our study reveals its crucial yet varied role as a predictor of co-occurring ED risk. The opposing effects of BMI in the two groups highlight the profound contextual differences between them and suggest that the relationship between body mass and eating pathology is not uniform and depends on the specific characteristics of the population under study.

Nicotine, as a psychoactive substance with stimulating effects and a simultaneous relaxing component that influences cognitive performance, mood modulation, and appetite reduction, gains a new perspective in the context of research on orthorexia [45]. The potential use of nicotine as a mechanism for coping with stress and controlling hunger in the general population served as a starting point for further analyses [46].

Particularly significant, in light of the multidimensional analysis, is the interpretation of the relationship between cigarette smoking and the risk of ON in Group 2. The unidimensional analysis suggested that a higher risk of ON occurs in non-smokers, which would seem consistent with the pursuit of a pure, pro-health lifestyle. This finding was consistent with the reports of Hyrnik et al., who also observed an association between non-smoking and orthorexia in a population of Polish adolescents [47]. However, after including other factors in the logistic regression model, especially the risk of ED, this relationship was completely reversed. Our model demonstrated that cigarette smoking is an independent predictor of a more than twofold higher risk of ON. This phenomenon may be an example of Simpson’s paradox, where the influence of a third variable—in this case, the risk of ED, which is negatively correlated with ON in this group—masked the true relationship [48]. A similar dependency was observed by Maghetti et al. in a study of Italian healthcare workers and by Fidan et al. in a study of medical students in Turkey [49,50]. These findings compel the rejection of a simplistic view of ON as a behavior exclusively consistent with pro-health attitudes and prompt the search for more complex explanations. Research on motivations and coping mechanisms may provide answers.

Weight control may also play a key role. A study by Oberle et al. showed that although non-smokers exhibit more orthorexia behaviors, among smokers, a higher level of ON was associated with smoking for weight control purposes [47]. Our result may therefore indicate that in the subgroup of individuals with ON in G2, smoking is not a manifestation of health ignorance but rather a tool subordinated to the overriding goal of appetite and body shape control—a feature common to the spectrum of eating disorders. Secondly, smoking may function as a non-adaptive mechanism for coping with stress and emotional tension. Research conducted by Wise et al. points to tobacco use as an affect regulation strategy in response to stress [46]. Considering that orthorexia is associated with high levels of anxiety, perfectionism, and rigorous rules, nicotine may be perceived by these individuals as a means of alleviating the psychological tension generated by their own obsessive dietary standards.

Furthermore, the two-dimensional model of orthorexia differentiates between “healthy orthorexia” (HO), associated with genuine concern for health, and the pathological “orthorexia nervosa” (ON). A study by Samaha et al. found that nicotine dependence was negatively correlated with HO but not with ON [43]. Our finding, linking smoking to the risk of ON, may therefore suggest that we are dealing with a more pathological form of the disorder in this group, where the pursuit of “health” is so distorted that it permits the use of harmful substances as tools to achieve goals related to eating and control.

A possible interpretation is that although smoking is objectively an unhealthy behavior, in this cohort, it may be associated with other, unmeasured factors that are, in turn, predictors of a higher score on the ORTO-15 scale. This result contradicts the simple thesis that ON always co-occurs with other pro-health behaviors and requires further, in-depth research.

The analysis of the role of gender in the context of ON risk yielded some of the most complex and unexpected results in our study. In the univariate analysis, no direct association was found between gender and ON risk, which is consistent with some literature that also indicates inconsistency or lack of gender differences in ON prevalence, although some studies in specific groups suggest different patterns [25,26,30,47].

This picture changed radically in the logistic regression models, where gender proved to be a strong predictor of eating disorder (ED) risk, but its effect was diametrically different in the two groups. In Group 1, in line with expectations and global epidemiology, men had an 84% lower odds of ED risk compared to women. This result confirms the well-established fact that women constitute a population at higher risk for developing classically understood eating disorders. In contrast, in Group 2, our model revealed that being male was associated with an almost 11-fold higher odds of ED risk. One possible explanation for this phenomenon is that the EAT-26 questionnaire, while designed to assess classic eating disorder (ED) symptoms, may have inadvertently captured symptoms of muscle dysmorphia or muscularity-oriented disordered eating (MODE) in this group, which are significantly more common in men. According to Thompson et al., MODE is characterized by behaviors such as consuming extremely high amounts of protein, rigidly restricting carbohydrates and fats, and using supplements and steroids to build muscle mass [51]. The obsessive thoughts and rigorous behaviors related to eating in MODE could have been interpreted by the men in Group 2 as applicable to the items on the EAT-26 scale, leading to high scores, despite their goal being muscularity rather than thinness. Therefore, this may be an artifact stemming from the specificity of the sample; men from Group 2, recruited from health and lifestyle forums, could have been a group with particular, unmeasured characteristics, such as high levels of muscularity-oriented body dissatisfaction, which may have influenced their responses on the EAT-26 questionnaire. Secondly, the result may reflect the growing, though still under-researched, problem of eating disorders among young men, who may present different, “male-specific” patterns of pathology not fully captured by traditional diagnostic tools. Finally, this is a product of a multivariable model that controls for other factors, which can reveal complex interactions not visible in simple comparisons.

Regardless of the underlying reason, this finding underscores that gender, while not a direct predictor of ON in our study, plays a crucial and context-dependent role in modulating the risk of co-occurring eating psychopathology. This highlights how divergent the risk profiles for men and women can be across different environments and warrants further investigation into the male-specific aspects of eating disorders.

Similarly, our study generally did not show a strong association between ON and age, education level, or place of residence, supporting the notion that ON may be a phenomenon relatively independent of basic demographic factors in the studied population of young adults [25,30].

Likewise, regarding the use of psychoactive substances (alcohol, drugs), our results indicating a lack of significant associations (apart from the specific link with non-smoking in Group 2) are consistent with other studies that also did not show a relationship between the overall severity of ON symptomatology and the status of using these substances, suggesting rather the importance of motivations for their use [31,47]. The available data therefore suggest that traditional demographic, social factors, or the general status of substance use have limited predictive value for ON risk in the studied population of young adults, which may be due to the specificity of the phenomenon itself or limitations of the measurement tools used [31].

An important issue that cannot be overlooked in the interpretation of the results is the absence of detailed socioeconomic data, such as income, and specific information on participants’ living arrangements. These factors could significantly influence dietary choices, access to specialized and often more expensive foods, and exposure to various lifestyle pressures. This limitation is particularly relevant when considering the paradoxical finding that Group 1, in which participants more frequently reported working, showed a lower risk of orthorexia than Group 2, which was more focused on academia. One potential explanation is that working individuals, especially at an early career stage, may have fewer financial resources and less time, which prevents them from following restrictive, costly diets characteristic of orthorexia. The role of financial status in the development of ON, however, appears complex and is not consistently supported in the literature. For instance, studies on Polish and Greek students by Dąbal and Gonidakis et al. found no significant relationship between financial status and ON symptoms [48,49]. Conversely, other studies suggest that greater financial resources may facilitate orthorexia behaviors. The latter view is strongly supported by a study on a Polish sample of adolescents conducted by Hyrnik et al., which found that the highest income correlated with the final address of orthorexia [47]. Furthermore, housing conditions may play a key role. Group 2 individuals, more embedded in the academic environment, may experience less direct financial pressure if they live with their families, which in turn may provide the stability needed to nurture orthorexia behaviors. However, research taking into account this variable is very limited or even unavailable.

The lack of these specific socioeconomic and housing data in our study is therefore a significant limitation that prevents a more precise understanding of the observed differences between groups.

## 5. Limitations of the Study

The findings of this study should be interpreted in the context of several important limitations that also highlight valuable directions for future research.

First, the study’s cross-sectional design precludes any inferences of causality. While we identified significant associations, such as the relationship between ON risk and ED risk, the temporal sequence of these phenomena cannot be determined from our data. Longitudinal studies are necessary to understand the developmental pathways of orthorexia and its dynamic interplay with other disorders. Furthermore, the recruitment was based on a non-probabilistic, convenience sampling method, which limits the generalizability of our findings to the broader Polish population of young adults. The differing sample sizes between the cohorts also affect the comparative statistical power, a factor that must be considered when interpreting the results. Finally, the entire study relied on self-report measures, which, while standard in this type of research, are susceptible to recall and social desirability biases.

Second, specific limitations related to the measurement instruments are noteworthy. Particular caution is warranted when interpreting results from the ORTO-15 questionnaire. The dramatically high prevalence of ON risk found in Group 2 and the low internal consistency of the scale observed in our cohorts reinforce existing critiques of its psychometric stability. The authors acknowledge that the ORTO-15 scale is subject to criticism in the scientific literature for its variable psychometric properties. Concerns include its variable internal consistency across different populations and the arbitrary nature of the cut-off point, which may lead to an overestimation of ON prevalence [31,32]. This tool may not adequately differentiate between pathological obsession and a normative, intense interest in healthy living, potentially leading to an overestimation of the phenomenon. Another significant measurement limitation is the assessment of depression via a single, non-validated question. While this method was used for brevity, it prevents a formal clinical diagnosis. A meta-analysis by Mitchell et al. indicated that such questions can possess acceptable sensitivity for screening but suffer from lower specificity, increasing the risk of false positives [15]. Therefore, the observed association with depression should be seen as a link to general psychological distress rather than a confirmed comorbidity. Similarly, the study did not include a measure of obsessive–compulsive symptoms (OCS). This omission was informed by previous findings from a large-scale Polish study, co-authored by one of the present paper’s authors, Łucka et al., which did not find a significant association between ON risk and OCS [19]. Nonetheless, the absence of a direct OCS measure in this specific cohort remains a limitation.

From an analytical perspective, the inclusion of multivariable analysis is a significant value-add to the current version of the study. Nevertheless, it should be noted that the logistic regression models were built based on the variables available in the collected dataset. Potential confounding factors, such as specific personality traits (e.g., perfectionism), socioeconomic status, or living arrangements, were not measured and could not be included in the models. This prevents full control over all variables that could influence ON and ED risk.

Finally, the study did not collect data on key socioeconomic factors, such as income, or on participants’ living arrangements (e.g., living with family, independently, or in student residences). These variables could significantly impact dietary choices, access to specialized foods, and exposure to different lifestyle pressures, and their absence limits the depth of our interpretation.

These limitations notwithstanding, this study provides a valuable cross-sectional snapshot and maps out a clear agenda for future, more methodologically robust research into the complex phenomenon of orthorexia nervosa.

## 6. Implications and Future Research Directions

The obtained results, despite the indicated limitations, carry significant clinical and research implications.

### 6.1. Implications for Clinicians

The confirmed strong association between ON and the eating disorder (ED) spectrum across all studied groups highlights the need to include orthorexia symptoms in the diagnostic and risk assessment processes in clinical practice. Clinicians and behavioral nutrition professionals should be vigilant for excessive focus on “healthy” eating, which may signal ON risk, especially when coupled with rigid dietary rules or obsessive food-related thoughts. This vigilance is further supported by meta-analytic findings indicating that prevention programs can be effective in reducing maladaptive eating attitudes and behaviors, with greater benefits observed in studies targeting participants at a relatively higher risk for developing an eating disorder [34].

The observed association with depression, although variable between groups and partially not statistically significant in G2, suggests the necessity of routine psychological assessment, including mood, in individuals with suspected ON. The drastically high prevalence of ON risk in Group 2 (76.8%) when applying the standard ORTO-15 threshold (<35 points) indicates that screening tools may lead to overdiagnosis in specific populations.

### 6.2. Implications for Researchers

This study points to several key directions for future research.

There is an urgent need to develop and validate more precise diagnostic tools for ON that would better differentiate healthy dietary concerns from pathological obsession and be more psychometrically stable than ORTO-15, especially given its low internal consistency in G1 and G2.

Further epidemiological studies using standardized methods are essential to better understand the actual prevalence of ON in different groups and to clarify the reasons for the observed discrepancies, such as the dramatic difference in prevalence between G1 and G2. Conducting longitudinal studies is crucial for understanding the dynamics of ON development, its place in the course of other mental disorders, and its long-term consequences.

It is also necessary to continue exploring the correlates of ON, including verifying the observed association with smoking in different populations, as well as thoroughly investigating the influence of personality traits (e.g., perfectionism), specific dietary patterns, the impact of social media on body image and food cravings, and experiences with weight stigma. Research should also explore the potential role of exposure to, and perception of, ultra-processed foods in motivating the rigid dietary rules seen in ON. This could lead to a better understanding of how the pursuit of “purity” in diet interacts with modern food environments and contributes to food addiction-like patterns of obsessive control, even in the absence of traditional addictive substances. Given that previous research on eating disorders and body image has often been limited to Western populations, and recognizing that studies in diverse cultural contexts can reveal unique and nuanced findings, future research on ON in Poland and other non-Western countries is particularly vital to advance our global understanding of these phenomena [50]. This includes exploring specific cultural factors that might influence body image ideals, food perceptions, and the manifestation of orthorexia tendencies.

In future analyses, quantitative studies should be complemented by qualitative research, which would allow for a better understanding of the subjective experiences of individuals with orthorexia tendencies.

## 7. Conclusions

This study confirms a complex, context-dependent relationship between Orthorexia Nervosa (ON) and other eating disorders among young adults in Poland. Multivariable analysis revealed that the nature of this relationship differs dramatically between the studied cohorts. In Group 1, ON risk was strongly and positively associated with both eating disorder (ED) risk and depression, aligning with classic eating pathology models. In stark contrast, the model for Group 2 revealed an inverse relationship between ON and ED risk, with smoking emerging as a significant independent predictor of ON.

These paradoxical findings, especially when considered alongside the extremely high prevalence of ON risk in Group 2 (76.8% vs. 26.5% in G1), critically underscore the limitations of the ORTO-15 questionnaire. The scale’s low internal consistency, confirmed in our study, suggests that in specific populations it may fail to distinguish pathology from a normative, intense interest in a healthy lifestyle *‘healthism’*, leading to confusing and contradictory results.

Our findings highlight the urgent need to develop and validate more precise diagnostic tools for ON. Further research is warranted to clarify its correlates in diverse populations, particularly regarding male-specific eating pathologies and the influence of socio-cultural factors, to better understand its place within the spectrum of mental disorders.

## Figures and Tables

**Table 1:** Key differentiating characteristics of the study groups.

Characteristic		Group 1 (n = 136)		Group 2 (n = 276)		Key Difference
		n	%	n	%	
Primary Occupational Profile	Working Only	39	28.7%	55	19.9%	G1 has a larger contingent in the workforce.
	Student Only	59	43.4%	152	55.1%	G2 is more concentrated in the academic setting.
Dietary Habits	Use of Special Diets	45	33.1%	150	54.3%	The prevalence of special diets is significantly higher in G2.

**Table 2:** Internal consistency of the ORTO-15 scale in the studied groups.

Reliability Characteristics	Group 1 (G1)	Group 2 (G2)
n (included in reliability analysis)	n = 136	n = 276
Cronbach’s Alpha coefficient (α) for ORTO-15	0.455	0.431

**Table 3:** Sociodemographic, anthropometric, and health-related characteristics of the study participants by group.

Characteristic		Group 1 (G1)	Group 2 (G2)
Number of participants (n)		136	276
Data collection method		Online survey	Online survey
Age (years)	Mean (±SD)	23.2 (±3.2)	23.3 (±4.7)
	Median (scope)	23.0 (17–39)	23.0 (17–54)
Sex (%)	Female	69.9% (n = 95)	73.6% (n = 203)
	Male	27.9% (n = 38)	24.6% (n = 68)
	Non-binary	2.2% (n = 3)	1.8% (n = 5)
Level of education (%)	Primary/Vocational	0.8% (n = 2)	0.8% (n = 2)
	Secondary	27.2% (n = 37)	27.2% (n = 75)
	Higher	71.3% (n = 97)	72.0% (n = 199)
Place of residence (%)	Village	11.8% (n = 16)	9.7% (n = 27)
	City up to 100 k	19.1% (n = 26)	19.6% (n = 54)
	City > 100 k	69.1% (n = 94)	70.7% (n = 195)
Occupational status (%)	Unemployed	5.1% (n = 7)	3.3% (n = 9)
	Working	28.7% (n = 39)	19.9% (n = 55)
	Working and studying	22.8% (n = 31)	21.7% (n = 60)
	Pupil/Student	43.4% (n = 59)	55.1% (n = 152)
BMI (kg/m^2^) (mean ± SD)		22.97 ± 4.56	22.97 ± 4.56
Category: BMI (%)	Underweight (including starvation and emaciation)	8.8% (n = 12)	7.3% (n = 20)
	Normal	72.1% (n = 98)	72.8% (n = 201)
	Overweight/Obesity (all grades)	19.1% (n = 26)	19.9% (n = 55)
Symptoms of depression (%)		33.8% (n = 46)	35.5% (n = 98)
Smoking (% of smokers)		24.3% (n = 33)	23.2% (n = 64)

**Table 4:** Prevalence of orthorexia nervosa (ON) risk and eating disorder (ED) risk in the study groups.

Risk (%)	Group 1 (G1) (n = 136)			Group 2 (G2) (n = 276)		
	n	%	95% CI	n	%	95% CI
ON Risk (ORTO-15 < 35)	36	26.5%	[19.7–34.3%]	212	76.8%	[71.5–81.4%]
ED Risk (EAT-26) ^1^	21	15.4%	[19.7–34.5%]	57	20.7%	[16.3–25.8%]

^1^ ED risk determined based on EAT-26 score.

**Table 5:** Multivariable logistic regression analysis for predictors of orthorexia nervosa (ON) risk.

Group	Predictor	b ^1^	S.E. ^2^	Wald ^3^	*df*	*p*	OR ^4^	95% CI ^5^ for OR
G1 (n = 136)	Depression	0.924	0.451	4.201	1	0.040	2.519	1.041–6.094
	EAT	2.431	0.555	19.186	1	<0.001	11.370	3.831–33.743
	Constant	−1.822	0.318	32.728	1	<0.001	0.162	N/A
G2 (n = 276)	Smoking	0.762	0.346	4.851	1	0.028	2.143	1.088–4.222
	EAT	−1.818	0.334	29.594	1	<0.001	0.162	0.084–0.313
	Constant	−0.092	0.287	0.103	1	0.748	0.912	N/A

^1^ b = unstandardized regression coefficient; ^2^ S.E. = Standard Error; ^3^ Wald = Wald chi-square statistic; ^4^ OR = Odds Ratio; ^5^ 95% CI = 95% Confidence Interval.

**Table 6:** Multivariable logistic regression analysis for predictors of eating disorder (ED) risk.

Group	Predictor	b ^1^	S.E. ^2^	Wald ^3^	*df*	*p*	OR ^4^	95% CI ^5^ for OR
G1 (n = 136)	Gender	−1.797	0.839	4.580	1	0.032	0.166	0.032–0.860
	BMI	0.109	0.054	4.129	1	0.042	1.115	1.004–1.239
	ON	2.497	0.579	18.600	1	<0.001	12.149	3.905–37793
	Constant	−4.956	1.369	13.107	1	<0.001	0.007	N/A
G2 (n = 276)	Gender	2.393	0.647	13.676	1	<0.001	10.943	3.079–38.894
	BMI	−0.103	0.039	6.879	1	0.009	0.902	0.836–0.974
	ON	−1.910	0.357	28.690	1	<0.001	0.148	0.074–0.298
	Constant	3.977	0.927	18.423	1	<0.001	53.379	3.977

^1^ b = unstandardized regression coefficient; ^2^ S.E. = Standard Error; ^3^ Wald = Wald chi-square statistic; ^4^ OR = Odds Ratio; ^5^ 95% CI = 95% Confidence Interval.

**Table 7:** Association between ON risk (ORTO-15 < 35) and depression in the study groups.

Group	Individuals with ON Risk in the ED Risk Group		Individuals with ON Risk in the No ED Risk Group		Chi-Square Test Statistic (χ^2^(*df*), *p*-Value, Cramer’s V)	Odds Ratio (OR) [95% CI]
	n	%	n	%		
Group 1 (G1) (n = 136)	15	71.4%	21	18.3%	χ^2^(1) = 25.790; *p* < 0.0011, V = 0.435	11.190 [3.883; 32.247]
Group 2 (G2) (n = 276)	27	47.4%	185	84.5%	χ^2^(1) = 35.0; *p* < 0.0011; V = 0.356	0.165 [0.088; 0.312]

**Table 8:** Association between ON risk (ORTO-15 < 35) and depression tendencies in the study groups.

Group	Association of ON with Depression Tendencies (χ^2^(*df*), *p*-Value; Cramer’s V)	ON Risk (Depression Tendencies YES)			ON Risk (Depression Tendencies NO)			Odds Ratio (OR) [95% CI]
		n	%	[95% CI]	n	%	[95% CI]	
Group 1 (G1) (n = 136)	χ^2^(1) = 3.927; *p* = 0.048; V = 0.170 (Significant)	17	37.0%	[24.5–51.4%]	19	21.1%	[13.9–30.7%]	2.191 [1.000; 4.798]
Group 2 (G2) (n = 276)	χ^2^(1) = 3.498; *p* = 0.061; V = 0.113 (Insignificant)	69	70.4%	[61.4–79.4%]	143	80.3%	[74.4–86.0%]	1.717 [0.971; 3.036]

**Table 9:** Association between ON risk (ORTO-15 < 35 points) and psychoactive substance use in the study groups.

Substance/Group		Group 1 (G1) (n = 136)	Group 2 (G2) (n = 276)
Tobacco (Smoking)	% ON+ (Smoking)	33.3%	67.2%
	% ON+ (Non-smoking)	24.3%	79.7%
Chi-square test statistic		χ^2^(1) = 1.054; *p* = 0.305; V = 0.088	χ^2^(1) = 4.333; *p* = 0.037; V = 0.125
Alcohol	% ON+ (Yes)	32.1%	77.3%
	% ON+ (No/occasionally)	25.0%	76.7%
Chi-square test statistic		χ^2^(1) = 0.583; *p* = 0.445; V = 0.065	χ^2^(1) = 0.006; *p* = 0.937; V = 0.005
Drugs (Use)	% ON+ (Yes/regular)	50.0%	61.1%
	% ON+ (No/tried)	24.2%	77.9%
Chi-square test statistic		χ^2^(1) = 3.744; *p* = 0.053; V = 0.166	χ^2^(1) = 2.665; *p* = 0.103; V = 0.098

**Table 10:** Correlation between ON risk and utilization of specialist mental healthcare (psychologist/psychiatrist) in the studied groups.

Characteristic	Group 1 (n = 136)		Group 2 (n = 276)	
Seeking psychological/psychiatric help	Yes	No	Yes	No
ON Risk: Yes (% within help category)	23 (29.9%)	13 (22.0%)	124 (75.2%)	88 (79.3%)
ON Risk: No (n (% within help category))	54 (70.1%)	46 (78.0%)	41 (24.8%)	23 (20.7%)
Total for help category (n)	77	59	165	111
Chi-square test statistic (χ^2^(*df* = 1))	1.054		0.635	
*p*-value	0.305		0.426	
Cramer’s V	0.088		0.048	
OR [95% CI]	1.5007 [0.687; 3.306]		1.265 [0.709; 2.257]	

## Data Availability

The original contributions presented in this study are included in the article. Further inquiries can be directed to the corresponding author.

## Abbreviations

The following abbreviations are used in this manuscript:

AN	Anorexia Nervosa
BMI	Body Mass Index
b	unstandardized regression coefficient
BN	Bulimia Nervosa
CI	Confidence Interval
EAT-26	Eating Attitudes Test-26
ED	Eating disorders
G1, G2	Group 1, Group 2
HO	Healthy Orthorexia
n	Number
N/A	Not available
OCD	Obsessive–compulsive disorder
ON	*Ortorexia Nervosa*
OR	Odds Ratio
ORTO-15	ORTO-15 Questionnaire
SD	Standard deviation
S.E	Standard Error
Wald	Wald chi-square statistic
